# Analysis of T Cell Subsets in Adult Primary/Idiopathic Minimal Change Disease: A Pilot Study

**DOI:** 10.1155/2017/3095425

**Published:** 2017-08-15

**Authors:** Francisco Salcido-Ochoa, Susan Swee-Shan Hue, Doreen Haase, Jason Chon Jun Choo, Nurhashikin Yusof, Reiko Lixiang Li, John Carson Allen, Jabed Iqbal, Alwin Hwai Liang Loh, Olaf Rotzschke

**Affiliations:** ^1^Tregs and HLA Research Force, Singapore General Hospital, The Academia, 20 College Road, Singapore 169856; ^2^Renal Medicine Department, Singapore General Hospital, The Academia, 20 College Road, Singapore 169856; ^3^Department of Pathology, National University Hospital, 5 Lower Kent Ridge Road, Singapore 119074; ^4^Kompetenznetz Vorhofflimmern e.V. (AFNET), Münster, Germany; ^5^Singapore Immunology Network (SIGN), Agency for Science, Technology and Research (A^⁎^STAR), Biopolis, Singapore; ^6^Department of Pathology and Laboratory Medicine, KK Women's and Children's Hospital, 100 Bukit Timah Road, Singapore 229899; ^7^Centre for Quantitative Medicine, Duke-NUS Graduate Medical School, The Academia, 20 College Road, Singapore 169856; ^8^Department of Pathology, Singapore General Hospital, The Academia, 20 College Road, Singapore 169856

## Abstract

**Aim:**

To characterise infiltrating T cells in kidneys and circulating lymphocyte subsets of adult patients with primary/idiopathic minimal change disease.

**Methods:**

In a cohort of 9 adult patients with primary/idiopathic minimal change recruited consecutively at disease onset, we characterized (1) infiltrating immune cells in the kidneys using immunohistochemistry and (2) circulating lymphocyte subsets using flow cytometry. As an exploratory analysis, association of the numbers and percentages of both kidney-infiltrating immune cells and the circulating lymphocyte subsets with kidney outcomes including deterioration of kidney function and proteinuria, as well as time to complete clinical remission up to 48 months of follow-up, was investigated.

**Results:**

In the recruited patients with primary/idiopathic minimal change disease, we observed (a) a dominance of infiltrating T helper 17 cells and cytotoxic cells, comprising cytotoxic T cells and natural killer cells, over Foxp3+ Treg cells in the renal interstitium; (b) an increase in the circulating total CD8+ T cells in peripheral blood; and (c) an association of some of these parameters with kidney function and proteinuria.

**Conclusions:**

In primary/idiopathic minimal change disease, a relative numerical dominance of effector over regulatory T cells can be observed in kidney tissue and peripheral blood. However, larger confirmatory studies are necessary.

## 1. Introduction

Primary minimal change disease (MCD) is a common cause of idiopathic nephrotic syndrome. Histopathologically, it is characterised by the apparent lack of abnormalities on light or immunofluorescence microscopy but effacement of foot processes on electron microscopy. It is believed that podocyte injury underlies the fundamental dysfunction in this disease. MCD is, therefore, also classified as a type of primary podocytopathy [1]. In this condition, disruption of the glomerular filtration barrier leads to increased glomerular permeability to proteins, causing severe urinary loss of proteins including albumin, immunoglobulins, complement, lipid-binding proteins, and clotting factors. The ensuing complications include hypoalbuminemia, generalised oedema, and malnutrition, as well as predisposition of patients to risk of infection, thrombotic events, and dyslipidemia.

Although the mechanisms underlying podocyte injury may include both nonimmunological factors and immunological factors [2, 3], they remain largely speculative and poorly understood. An interesting breakthrough in the science of MCD was finding an association of high serum levels and overexpression on podocytes of a sialic acid-deficient form of the glycoprotein angiopoietin-like 4 with several clinical, functional, and histopathological features of human and murine MCD, including the severity of proteinuria, effacement of podocyte foot processes, loss of glomerular basement membrane charge, and sensitivity to corticosteroids therapy [4]. Auspiciously, the treatment with N-acetyl-D-mannosamine (a sialic acid precursor) significantly improved proteinuria in the affected animals. On the other hand, immune factors, in particular T cell dysfunction and the release of circulating glomerular permeability factors (which are putative cytokines), are among the most commonly implicated contributing immunological alterations behind the occurrence of MCD [5–7]. Given the well-known role of Foxp3^+^ regulatory T (Treg) cells as the master moderators of immune responses [8, 9], it is conceivable that a dysfunction of Treg cell biological functions or a numerical deficiency may contribute significantly to the pathogenesis of MCD.

Foxp3^+^ Treg cells play a central role in the maintenance of immune homeostasis. They moderate ongoing immune responses by controlling the activation, proliferation, and effector functions of various cells of the innate defence and the immune systems. Of note, they can be divided into two main subsets, thymic and peripheral Foxp3^+^ Treg cells, having different origins as well as distinct and complementary functions in both physiological and pathological immune situations [10–12]. Thymic Foxp3^+^ Treg cells acquire their immunoregulatory capacities in the thymus while being selected by the self-major histocompatibility complex molecules and self-antigens present in the thymus. The role of thymic Foxp3^+^ Treg cells in the periphery, therefore, appears to be maintenance of immune tolerance through modulation of activation, differentiation, and proliferation of potential autoreactive T cell clones [13, 14]. On the other hand, peripheral Foxp3^+^ Treg cells derive from stimulation of naïve T cells with foreign antigens or altered self-antigens in secondary lymphoid organs and inflamed tissues [9]. The generation of peripheral Foxp3^+^ Treg cells is driven by the concomitant activation of effector T (Teff) cells, which serve as a source of IL-2 and other permissive cytokines that are necessary for their development [15, 16]. Thus, the role of peripheral Foxp3^+^ Treg cells appears to be the control of ongoing immune responses to foreign antigens and self-antigens not expressed in the thymus, thereby preventing inappropriate inflammatory responses and autoimmune disorders triggered by inflammation or infection where T helper (Th)17, Th1, and cytotoxic T cells (CTL) are believed to play an active role [11, 13, 14, 17]. A dysfunction of Foxp3^+^ Treg cells, encompassing both numerical and functional imbalance between Foxp3^+^ Treg cells and Teff cells, may lead to a disturbance of immune homeostasis that potentially contributes to disease pathogenesis in MCD.

Indeed, previous studies have suggested such dysfunction of Foxp3^+^ Treg cells in the pathogenesis of primary MCD. A study performed on 38 children with primary MCD or focal segmental glomerulosclerosis reported a lesser infiltration of Foxp3^+^ T cells in affected patients compared to controls [18]. Another study performed on 21 adults with primary MCD demonstrated decreased suppressive capacity of Foxp3^+^ Treg cells, although patients had similar numbers of circulating Foxp3^+^ Treg cells in comparison with controls [19]. In another report, 21 children with steroid-resistant nephrotic syndrome had increased ratios of circulating interferon- (IFN-) gamma-secreting Th1 cells and IL-4-secreting Th2 cells over Foxp3^+^ Treg cells when compared to 22 children with steroid-sensitive nephrotic syndrome in clinical remission and 14 healthy controls [20]. The interplay between Th17 cells and Foxp3^+^ Treg cells has also been reported in two studies. One study showed an increase in circulating Th17 cells over Treg cells in 25 adult patients with primary MCD when compared to healthy controls, which also correlated with severity of proteinuria [21]. The other study performed on 36 children with idiopathic nephrotic syndrome demonstrated an increase in circulating Th17 cells over Treg cells when compared to healthy controls, which was associated with an increase in intrarenal IL-17 expression [22].

Our primary aim was to confirm whether a similar immune imbalance of T cell subsets is a feature of primary MCD in our population. We focused primarily on the numerical balance of Th17 and cytotoxic cells [comprising CTL and natural killer (NK) cells] over the Foxp3^+^ Treg subset in kidney tissue by immunohistochemistry, using their relative ratio as an arbitrary measure of such balance. We have described our tissue results as a case series due to the lack of normal kidney tissue for comparison. We also detected and quantified different Teff cells in the peripheral blood of patients using flow cytometry but focused our main analysis on Th17 cells, CD8^+^ T cells (a surrogate phenotype for CTL), and Foxp3^+^ Treg cells. We compared our findings in the peripheral blood of these patients to our observations in healthy controls. Finally, we explored correlations between our chosen immune parameters and clinical outcomes, including deterioration of kidney function and proteinuria, as well as time to complete clinical remission. Therefore, this is a pilot descriptive study reporting the observed frequencies of different immune cell subsets in kidney tissue and peripheral blood of MCD patients and their association with clinical outcomes.

## 2. Material and Methods

### 2.1. Study Design and Patients

This was a single-centre prospective study in a cohort of 9 consecutive patients aged 21–80 years with new-onset biopsy proven MCD thought to be idiopathic in nature. Patients were recruited at the Department of Renal Medicine of the Singapore General Hospital (SGH) between 1 January 2012 and 31 March 2014 at the time when native kidney biopsy was performed for the investigation of new-onset nephrotic syndrome and followed for up to 48 months for outcomes analysis. Healthy individuals were recruited to serve as a control group for many of the comparisons in the peripheral blood studies. Signed informed consent was taken from all participants and our protocol was approved by our Centralised Institutional Review Board (Approval number 2009/672/E).

### 2.2. Exclusion Criteria

HIV infection, history of haematological malignancies, children, pregnant women, poor cognitive capacity, prisoners, and inability to understand the research protocol and give consent were the exclusion criteria.

### 2.3. Clinical Data

Baseline demographic and clinical characteristics were retrieved from patient hard-copy case notes and electronic medical records. Use and type of immunosuppressants prescribed were also recorded.

### 2.4. Routine Laboratory Investigations

Serum creatinine was measured and proteinuria was quantified by means of either the urine protein to creatinine ratio or a 24-hour collection of total urinary protein. Calculated estimated glomerular filtration rate (GFR) was obtained through the modified diet in renal disease (MDRD) equation. All laboratory parameters were retrieved prospectively from electronic medical records at 6-month intervals from the time of native kidney biopsy up to 48 months of follow-up. All laboratory investigations were conducted at the SGH's clinical laboratory, which is accredited by the College of American Pathologists.

### 2.5. T Cell Subset and B Cell Detection in Native Kidney Biopsies by Immunohistochemistry

Immunohistochemistry for detection of T cell subsets and B cells in native kidney tissue biopsies was performed at the laboratories in both the Renal Department and Department of Pathology of the SGH. In brief, sections prepared from formalin-fixed and paraffin-embedded kidney tissue specimens were stained with monoclonal antibodies conjugated with either horseradish peroxidase or alkaline phosphatase and directed against different phenotypic markers, including CD4 (used as a surrogate marker of CD4^+^ T cells), CD8 (used as marker of CD8^+^ T cells), CD19 (used as marker of B cells), IL-17 (used as marker of IL17-secreting Th17 cells), granzyme B (a marker of cytotoxic cells, comprising both CTL and NK cells), and Foxp3 (used as a surrogate marker of Treg cells) using an automatic stainer machine. The binding of the different antibodies onto the kidney tissue samples was revealed using the respective chromogenic substrates for those enzymes. Isotype-matched antibodies were used as negative controls. Tonsil tissue served as positive control. Staining was visualized and measured by light microscopy semiquantitatively as percentage of infiltration for CD4^+^, CD8^+^, and CD19^+^ cells and expressed as number of cells per mm^2^ of kidney biopsy (cell density) by adjusting to biopsy surface area measured by Olympus CellSens software for Foxp3^+^, IL-17^+^, or granzyme B^+^ cells.

### 2.6. Phenotypic Analysis of T Cell Subsets by Flow Cytometry

The quantification of total CD3^+^ T cells, CD4^+^ T cells, CD8^+^ T cells, and CD19^+^ B cells in peripheral whole blood of patients and healthy controls was performed by flow cytometry using a BD LSR II machine available at the haematology laboratory of the SGH, using fluorochrome-labelled monoclonal antibodies against CD45, CD3, CD4, CD8, and CD19. The quantification of CD4^+^CD25^+^Foxp3^+^ Treg cells, CD4^+^CD161^+^ Th17 cells, CD4^+^CD45RA^−^ memory T cells, CD4^+^CD45RA^+^ naïve T cells, CD8^+^CD45RA^−^ memory T cells, CD8^+^CD45RA^+^ naïve T cells, CD3^+^CD56^+^ natural killer T (NKT) cells, CD3^+^TCRd1^+^ gamma-delta T cells, CD3^+^TCRd2^+^ gamma-delta T cells, CD4^−^CD161^+^TCRa7.2^+^ mucosal-associated invariant T (MAIT) cells, and CD3^−^CD56^+^ natural killer (NK) cells in stored peripheral blood mononuclear cells of patients and healthy controls was performed by flow cytometry using a BD LSR II machine available at the Singapore Immunology Network, using fluorochrome-labelled monoclonal antibodies against different phenotypic markers including CCR6, CD3, CD4, CD5, CD8, CD14, CD19, CD25, CD27, CD28, CD39, CD45RA, CD45RO, CD49d, CD56, CD127, CD152, CD161, GARP, TCRa7.2, TCRd1, and TCRd2. All the stained cells were acquired in a 20-colour flow cytometer, and the results were analysed using FlowJo v9.5 software. For compensation and calibration purposes, commercially available fluorochrome-labelled beads were used as controls. Percentages of marker-expressing cells were reported and adjusted as percentage of the lymphocyte counts. Absolute numbers of these T cell subsets were obtained by adjusting the percentages to the lymphocyte counts obtained through measuring the full blood count in the SGH's clinical laboratory.

### 2.7. Statistical Analysis

Being an exploratory, descriptive study in consecutively recruited patients during the allowed recruitment period, sample size was not calculated. Descriptive statistics were used to display our results obtained from our immunohistochemical investigations. No comparison with healthy control kidney tissue was possible. The different lymphocyte and T cell subset percentages and ratios in peripheral blood were compared between the patients with primary MCD and the healthy controls using the Mann–Whitney *U* test. Spearman correlation was used to assess strength of association of lymphocyte and T cell subset percentages and numbers in kidney tissue and peripheral blood with different clinical outcomes, including changes in serum creatinine, MDRD GFR, and proteinuria, as well as time to complete clinical remission, doubling of creatinine, and need for dialysis. All analyses were performed using SAS V9.3 software (SAS Inc., Cary, NC, USA).

## 3. Results

Nine patients with primary MCD were included in this study. All had their native kidney biopsy specimens tested by immunohistochemistry for detection of different T cell subsets and B cells. The obtaining of normal kidney tissue (or a surrogate tissue for normality) as control for our immunohistochemical studies was not possible. Eight patients provided blood sample for measurement of different T cell subsets in peripheral blood by flow cytometry. As many as twelve healthy individuals served as controls for our immune cell detection in peripheral blood by flow cytometry. Baseline demographic and clinical characteristics of recruited patients are shown in Table 1. Most patients were young Chinese males with full-blown nephrotic syndrome and not taking steroids or immunosuppression at the time of biopsy. Detailed clinical parameters for each patient are presented in Table 2.

**Figure 1:**
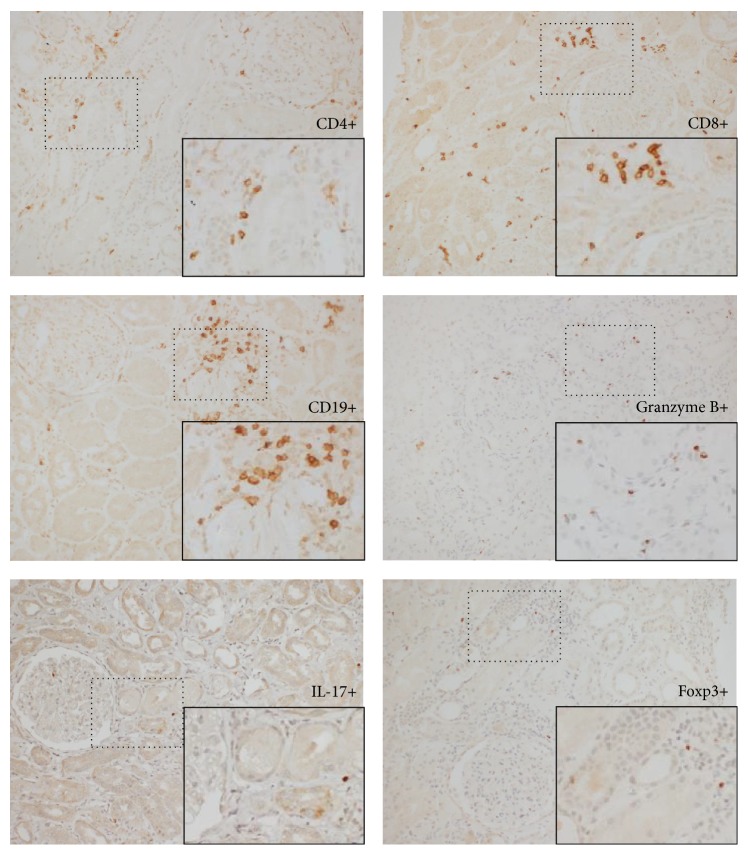
Detection of different lymphocyte and T cell subsets infiltrating kidney tissue in a patient with primary MCD with a mild-moderate infiltrate, using antibodies to CD4, CD8, CD19, granzyme B, IL-17, and Foxp3 as labelled on the pictures. An area with a significant infiltrate was selected for display purposes (200x magnification). The* inlets* on the lower right corner of each single-staining slide show a higher magnification (400x) of selected regions* (small squares)* to enhance visualization of marker-positive cells [immunohistochemistry staining using peroxidase conjugated antibodies].

**Figure 2:**
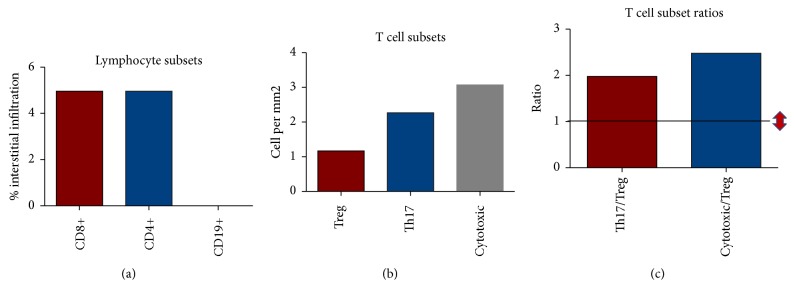
(a) Percentage of cortex infiltration of CD8^+^ T cells, CD4^+^ T cells, and CD19^+^ B cells in kidney tissue of 9 patients with primary MCD. (b) Cell densities of the infiltrating Foxp3^+^ Treg cells, IL-17^+^ Th17 cells, and granzyme B^+^ cytotoxic cells in kidney tissue of these primary MCD patients. (c) Ratios of the cell densities of Th17 cells over Foxp3^+^ Treg cells and cytotoxic cells over Treg cells, infiltrating kidney tissue of primary MCD patients (the horizontal* black line* indicates the arbitrary ratio reference value of 1; the* red arrows* indicate the direction of the ratios, increased or decreased). Cells were detected by immunohistochemistry, and the median percentages, cell densities, and their ratios are reported.

Figure 1 shows a phenotypic panel (by immunohistochemistry) of several T cell subsets and B cells in a patient with primary MCD. The pictures were taken at an area of moderate infiltration of both T and B cells for demonstration of our detection capability as the infiltrate was scarce and the overall staining was discrete in most patients, but sufficient for subset identification and quantification. The cell infiltrate in MCD patients was composed mainly of CD4^+^ and CD8^+^ T cells with a small population of B cells. The median percentage of infiltration of CD4^+^ T cells, CD8^+^ T cells, and CD19^+^ B cells, and the median cell density (cells per square millimeter of kidney biopsy) of the IL-17^+^ cells (Th17 cells), granzyme B^+^ cytotoxic cells (comprising both CTL and NK cells), and Foxp3^+^ cells (Treg cells) is plotted in Figure 2. We observed higher cell densities of Th17 cells and cytotoxic cells when compared to Foxp3^+^ Treg cells. As a consequence, the ratios of Th17 cells over Foxp3^+^ Treg cells and cytotoxic cells over Foxp3^+^ Treg cells, an arbitrary measurement of the numerical balance between these subsets, were increased. We had no access to normal kidney tissue for comparison of our findings and to investigate whether our observed frequencies and ratios were different to the ones observed in healthy individuals.

**Figure 3:**
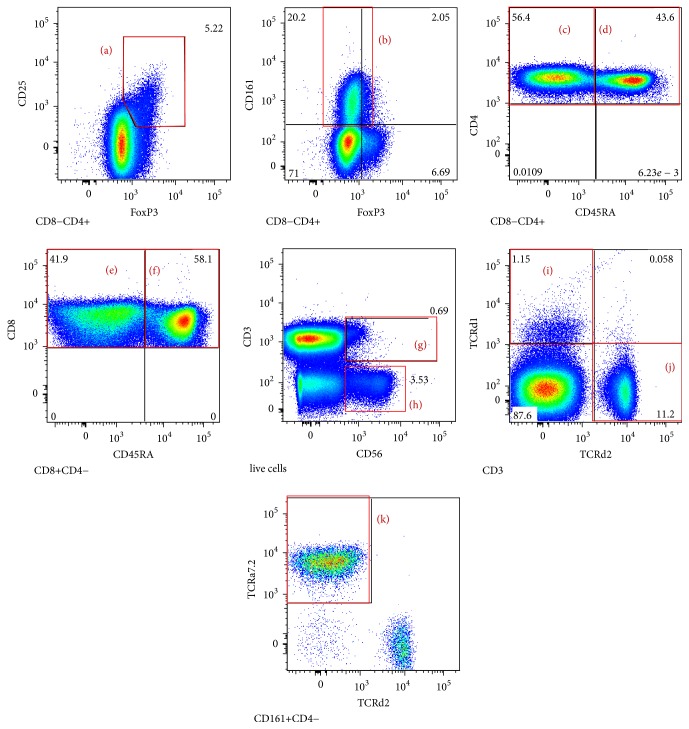
Representative surrogate T cell subsets in peripheral blood of a patient with primary MCD. CD4^+^CD25^+^Foxp3^+^ Treg cells (a), CD4^+^CD161^+^ Th17 cells (b), CD4^+^CD45RA^−^ memory T cells (c), CD4^+^CD45RA^+^ naïve T cells (d), CD8^+^CD45RA^−^ memory T cells (e), CD8^+^CD45RA^+^ naïve T cells (f), CD3^+^CD56^+^ NKT cells (g), CD3^−^CD56^+^ NK cells (h), CD3^+^TCRd1^+^ gamma-delta T cells (i), CD3^+^TCRd2^+^ gamma-delta T cells (j), and CD4^−^CD161^+^TCRa7.2^+^ MAIT cells (k) were detected by 20-colour flow cytometry, using the appropriate antibodies as described in the methods section.

**Figure 4:**
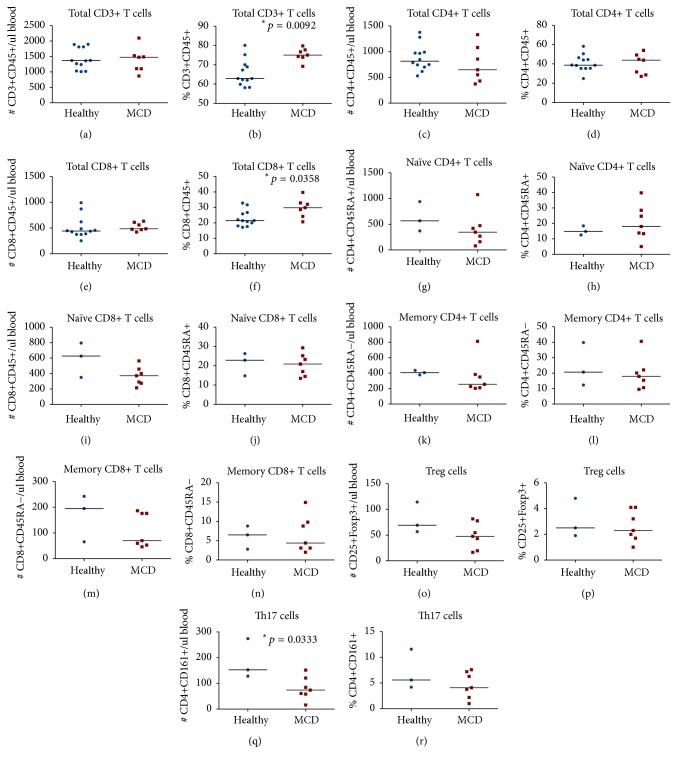
Comparative circulating cell numbers per microlitre of blood and comparative percentages from total lymphocytes of circulating total CD3^+^ T cells (a, b), total CD4^+^ T cells (c, d), naïve CD4^+^CD45RA^+^ T cells (g, h), memory CD4^+^CD45RA^−^ T cells (k, l), total CD8^+^ T cells (e, f), naïve CD8^+^CD45RA^+^ T cells (i, j), memory CD8^+^CD45RA^−^ T cells (m, n), CD4^+^CD25^+^Foxp3^+^ Treg cells (o, p), and CD4^+^CD161^+^ Th17 cells (q, r) between 7 primary MCD patients and up to 12 healthy controls. Cells were detected by 20-colour fluorescence-activated flow cytometry. The median cell numbers are represented by the horizontal lines. The* asterisks* indicate that the comparisons were statistically significant and the respective *p* value is given.

**Figure 5:**
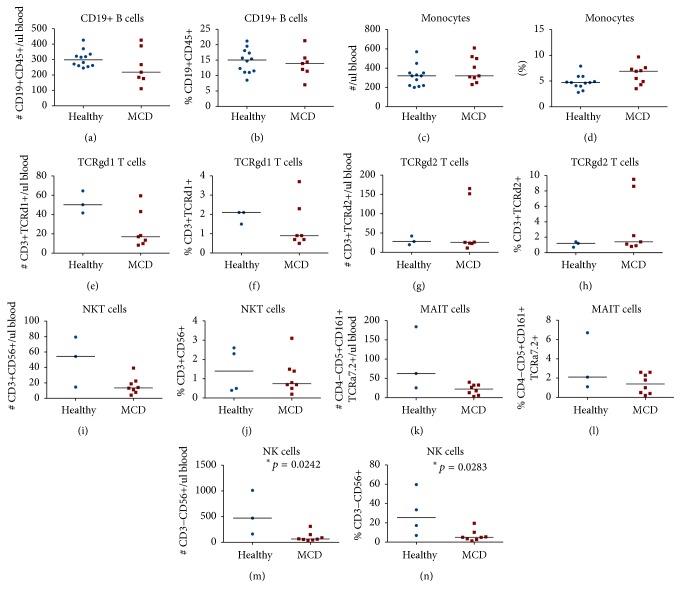
Comparative circulating cell numbers per microlitre of blood and comparative percentages from total lymphocytes of circulating CD19+ B cells (a, b), monocytes (c, d), CD3^+^TCRd1^+^ gamma-delta T cells (e, f), CD3^+^TCRd2^+^ gamma-delta T cells (g, h), CD3^+^CD56^+^ NKT cells (i, j), CD4^−^CD161^+^TCRa7.2^+^ MAIT cells (k, l), and CD3^−^CD56^+^ NK cells (m, n) between up to 8 primary MCD patients and up to 12 healthy controls. Cells were detected by 20-colour fluorescence-activated flow cytometry. The median cell numbers are represented by the horizontal lines. The* asterisks* indicate that the comparisons were statistically significant and the respective *p* value is given.

**Figure 6:**
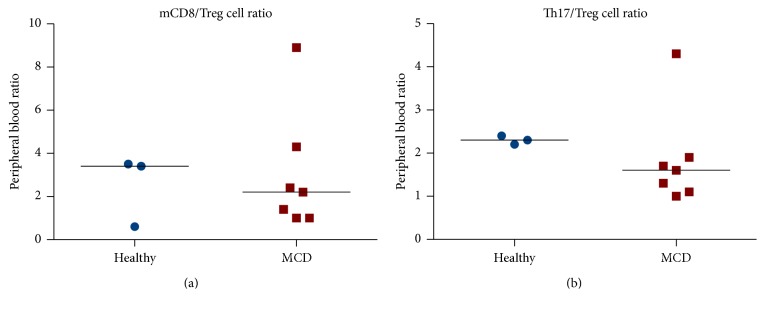
Comparative ratios of circulating Th17 cells over Treg cells (a) and memory CD8^+^ T cells over Treg cells (b) between 7 primary MCD patients and 3 healthy controls. Cells were detected by 20-colour fluorescence-activated flow cytometry. The median ratios are represented by the horizontal lines. The comparisons did not reach statistical significance.

In peripheral blood we measured the percentage (from the lymphocyte count) and the absolute numbers per microlitre of blood of circulating CD3^+^ T cells, CD4^+^ T cells, CD8^+^ T cells, CD19^+^ B cells, and monocytes, as well as CD4^+^CD25^+^Foxp3^+^ Treg cells, CD4^+^CD161^+^ Th17 cells, CD4^+^CD45RA^−^ memory T cells, CD4^+^CD45RA^+^ naïve T cells, CD8^+^CD45RA^−^ memory T cells, CD8^+^CD45RA^+^ naïve T cells, CD3^+^CD56^+^ NKT cells, CD3^+^TCRd1^+^ gamma-delta T cells, CD3^+^TCRd2^+^ gamma-delta T cells, CD4^−^CD161^+^TCRa7.2^+^ MAIT cells, and CD3^−^CD56^+^ NK cells.  Figure 3 illustrates some of the T cell subsets detected using multicolour flow cytometry in a representative patient with primary MCD. In peripheral blood, we found significantly higher percentages of total CD3^+^ T cells (*p* = 0.0092) and of total CD8^+^ T cells (*p* = 0.0358) in primary MCD patients when compared to healthy controls (Figures 4(b) and 4(f)), but there was no significant difference in the percentages or numbers of naïve and memory CD8^+^ T cells between the two groups. Foxp3^+^ Treg cells were not significantly reduced in primary MCD patients in comparison to the healthy controls (Figures 4(o) and 4(p)). On the other hand, the number of Th17 cells was significantly reduced in primary MCD patients in comparison with controls (*p* = 0.0333; Figure 4(q)). There were no statistically significant differences in the other T cell subsets or immune cells studied between primary MCD and the healthy controls (Figure 5), except for the finding of lower circulating NK cells in patients with primary MCD. Finally, there were no differences in the calculated ratios of circulating Th17 cells over Foxp3^+^ Treg cells or circulating memory CD8^+^ T cells over Foxp3^+^ Treg cells (Figure 6).

**Figure 7:**
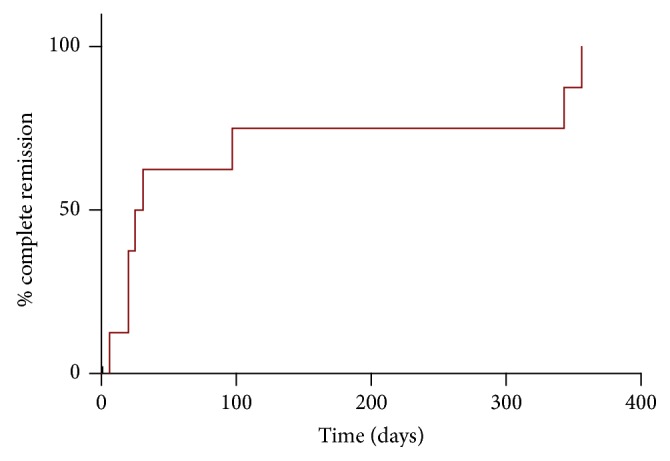
Time to complete remission of the 9 patients with primary MCD. The median time to complete remission was 28 days.

**Figure 8:**
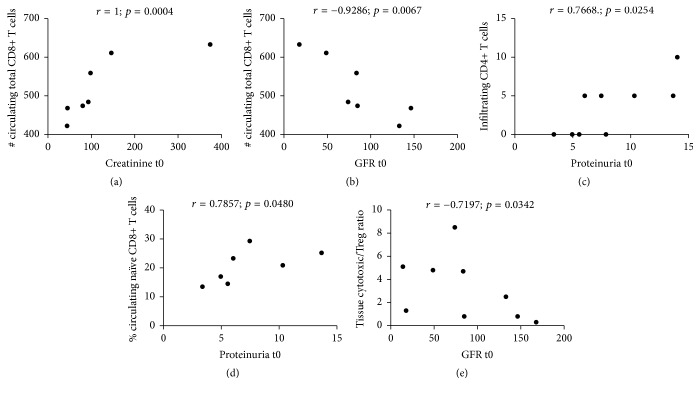
Selected correlation plots between lymphocyte and T cell subsets and clinical outcomes at disease onset in primary MCD. This figure is supplementary to Table 3, where the complete list of statistically significant correlations between outcomes and other disease subsets is found. Only the comparisons that were (1) statistically significant, (2) related to outcomes at disease onset* (t0)*, (3) related to our original hypothesis that the immune balance in MCD is tilted towards a dominance of Th17 and cytotoxic cells over the Foxp3^+^ Treg subset, and (4) discussed in the text are plotted here for visual reference.* Correlations plotted*: number of circulating CD8^+^ T cells with serum creatinine (a); number of circulating CD8^+^ T cells with MDRD GFR (b); percentage of kidney tissue infiltrating CD4^+^ T cells with proteinuria (c); percentage of circulating naïve CD8^+^ T cells with proteinuria (d); and ratio of tissue cytotoxic over Foxp3^+^ Treg cells with MDRD GFR (e).

We correlated selected immune cellular parameters with clinical outcomes to address any potential clinical meaning, as an exploratory analysis.  Table 3 shows the statistically significant correlations found between the circulating and infiltrating immune subsets with serum creatinine, MDRD GFR, and proteinuria (at different time points during follow-up), as well as time to complete clinical remission in our exploratory analyses. Complete clinical remission was achieved in these patients with a median time of 28 days (Figure 7) with subsequent stabilisation of serum creatinine. No patient had a doubling of creatinine or underwent dialysis. In relationship with our original hypothesis, at disease onset, the number of circulating CD8^+^ T cells correlated positively with serum creatinine (Figure 8(a)) and negatively with MDRD GFR (Figure 8(b)), the percentage of kidney tissue infiltrating CD4^+^ T cells (Figure 8(c)) and the percentage of circulating naïve CD8^+^ T cells (Figure 8(d)) correlated positively with proteinuria, and the ratio of tissue cytotoxic over Foxp3^+^ Treg cells correlated negatively with MDRD GFR (Figure 8(e)).

## 4. Discussion

Although the immunopathogenesis of primary MCD is complex and remains largely nondeciphered, it has been proposed that disturbances in the immune balance between Teff cells and Foxp3^+^ Treg cells play a potential pathogenic role [23]. This pilot study was conducted to describe the relative frequencies of different Teff cells and Foxp3^+^ Treg cells, in both tissue and peripheral blood, as an arbitrary measurement of their immune balance in primary MCD. Despite the small sample size, its main strength is the combining of tissue data with detailed phenotypic analysis of different immune cell subsets in peripheral blood as well as with clinical parameters. To the best of our knowledge, this has not been attempted in previous studies.

In tissue, we found the overall T cell infiltration into the kidney interstitium of patients with primary MCD to be minimal (Figure 2). Our finding that kidney interstitial infiltrates of these primary MCD patients were composed mainly of T cells rather than B cells is in keeping with the notion that primary MCD is fundamentally a T cell disorder. In addition, there appeared to be a relative deficiency of Foxp3^+^ Treg cells over Th17 cells and cytotoxic cells. Our study showed that the ratios of Th17 cells over Foxp3^+^ Treg cells, as well as cytotoxic cells over Foxp3^+^ Treg cells, were elevated (arbitrarily deciding a ratio above 1). Although ideally infiltrates in normal healthy subjects would be used for comparison, it was ethically unachievable in our descriptive study. Other studies have compared their observations with those from living donor kidney tissue or patients with thin-membrane disease [18, 24]. At the SGH, no biopsy is obtained preimplantation in living donors, and none of the patients recruited in our parallel studies had thin-membrane disease as diagnosis. In those studies, healthy or “normal” controls were found also to have a scarce cellular infiltrate, which could be or not comparable to that of our patients with MCD. As a consequence, in this paper, we are mainly describing and partially dissecting phenotypically the observed infiltrate and reporting their relative frequencies, which is a commonly encountered way to assess immune balance. But we do accept that this is an arbitrary and imperfect way to assess immune balance, especially when in vivo a complex functional and numerical interplay of the different immune arms activated or dysfunctional in several human disorders, rather than just a numerical balance, is taking place. However, that interplay can only be assessed with a multidimensional interrogation of the functional phenotypes present in disease states, as well as a detailed assessment of the hierarchy of involved cellular and molecular interactions, but this was beyond the scope and reach of our study design and aims. In addition, our findings are consistent with published data that demonstrates a possible deficiency of tissue-infiltrating Foxp3^+^ Treg cells in primary MCD [18]. In addition, in a study reported in 2002, gene expression for granzyme B was detected intrarenally in 2 of 7 children with minimal change disease [25].

Concordant with published observations [19], our primary MCD patients displayed similar numbers of circulating Foxp3^+^ Treg cells in comparison with healthy controls. However, counter to our hypothesis and the two reports previously mentioned [21, 22], we did not observe an increased ratio of circulating Th17 cells over Foxp3^+^ Treg cells in patients with primary MCD in comparison to healthy controls. It is possible that these discrepant observations were due to the different technological approaches to detect Th17 cells. We used CD161 as a surrogate marker for Th17 cells (on CD3^+^CD4^+^ T cells) and we observed a decreased number of circulating Th17 cells in the patients with primary MCD in comparison to healthy controls. On the contrary, those authors based their detection on intracytoplasmic IL-17 expression, which indeed could be a more reliable marker. However, in those studies, the detection of IL-17-expressing cells required in vitro stimulation of T cells, which in our opinion (based on our unpublished observations with CyTOF mass cytometry in a different project in kidney transplantation) would likely overrepresent the actual frequencies of this subpopulation in vivo. Irrespective of this possibility, our tissue and peripheral blood results mirror themselves as we observed an apparent reduction of both infiltrating and circulating Th17 cells, implying that our observation not necessarily reflects technological difference but possibly a characteristic of this condition. Once again this is not conclusive as we lack a tissue control for comparison.

A technical limitation worth mentioning in our study, inherent of single-staining immunohistochemistry using surrogate cell markers, is the assumption that all the CD4^+^ expressing cells were CD4^+^ T cells, all the Foxp3^+^ cells were Treg cells, and all the granzyme B^+^ cells were CTL and NK cells. Immunofluorescence could have circumvented this limitation, which can be considered in subsequent grant applications.

It was interesting to observe some degree of association of our immune parameters with clinical features at presentation and with disease outcomes. Most of our patients achieved complete clinical remission promptly after initiation of steroid therapy and none doubled serum creatinine levels or required dialysis. Therefore, we focused our clinical outcomes analysis on levels of proteinuria and kidney function at disease onset and on time to clinical remission. In tune with our overall hypothesis, we found associations of infiltrating CD4^+^ T cells and circulating CD8^+^ T cells with higher proteinuria at onset and also correlation of circulating CD8^+^ T cells and the infiltration ratio of cytotoxic cells over Foxp3^+^ Treg cells with worse kidney function at presentation. Overall, these observations appear to support the long-standing notion that primary MCD could be a T cell-mediated disorder or that T cell dysfunction could play a significant role in its pathogenesis. However, these observations arose from exploratory analyses in a limited number of cases and should be taken with reserve. Nevertheless, we are reporting them for potential hypothesis-generation purposes rather than to suggest conclusions from them.

Overall, our results show that a numerical imbalance of different immune subsets, in particular a deficiency of Foxp3^+^ Treg cells or a dominance of effector Th17 cells and cytotoxic cells, could be a characteristic of primary MCD. However, larger confirmatory studies looking into physiopathogenic mechanisms are necessary.

## 5. Conclusion

Primary MCD is an important cause of idiopathic nephrotic syndrome in adults. Different immune subsets can be detected in the renal interstitium of MCD patients, where a relative numerical abundance of Th17 cells and cytotoxic cells over Foxp3^+^ Treg cells appears to be a characteristic of this condition. Although our observations are preliminary, they enhance the current limited knowledge on this topic, and although our paper does not provide functional data, our findings could incite further detailed studies to assess more mechanistically the so-called immune imbalance between Foxp3^+^ Treg and Teff cells and its contribution in the pathogenesis of podocyte injury in primary MCD. A better understanding of the immunopathogenesis of primary MCD is necessary for the improvement of our current diagnostic armamentarium and the development of more targeted immunotherapy, which might potentially involve T cell subset modulation.

## Figures and Tables

**Table 1:** Baseline clinical and demographic characteristics of the patients with primary MCD.

*n*	9
Age (years)^*∗*^	31.4
Male sex (#)	6/9
Race, Chinese (#)	8/9
Diabetes mellitus (#)	1/9
GFR at biopsy (ml/min/1.73 m2)^*∗*^	83.6
Proteinuria at biopsy (g/day)^*∗*^	7.46
Albumin (g/L)^*∗*^	15
Steroids use at biopsy (#)	2/9
MMF or AZA use at biopsy (#)	0/9
Cytotoxics use at biopsy (#)	0/9

AZA: azathioprine; GFR: glomerular filtration rate; MMF: mycophenolate mofetil.  ^*∗*^Results reported as median values.

**Table 2:** Summary of the clinical characteristics and outcomes of the patients with primary MCD.

Patient	Age/sex	Race	Diabetes mellitus	GFR at onset	GFR last follow-up	Proteinuria at onset	Proteinuria last follow-up	Albumin at onset	Steroids at biopsy	Days to complete remission	Total days of follow-up
1	25/F	Chinese	No	167.8	132.2	7.9	0.16	17	No	356	1385
2	52/M	Chinese	No	73.9	93.4	5.6	0.1	9.0	No	25.0	1320
3	31/M	Indian	No	48.7	96.7	10.3	0.05	13	No	20	1364
4	21/M	Chinese	No	17.7	77.9	7.5	0.1	15.0	No	97	1256
5	47/F	Chinese	No	132.9	132.9	13.7	13.7	14.0	No	UNK	1
6	21/F	Chinese	No	83.6	84.4	5.0	0.1	15.0	Yes	6	1197
7	25/F	Chinese	No	146.4	115.4	6.0	0.0	17.0	Yes	20.0	298
8	62/M	Chinese	No	84.8	57.3	3.4	0.3	30.0	No	31	1102
9	65/M	Chinese	Yes	14.0	67.6	14.0	14.4	20.0	No	343	925

F: female; GFR: glomerular filtration rate; M: male; MCD: minimal change disease; UNK: unknown due to loss of follow-up.

**Table 3:** Correlation between lymphocyte and T cell subsets and clinical outcomes in primary MCD.

Immune cell parameter	versus	Clinical outcome	*R*	*p* value
Circulating total CD8+ T cells (#)		Serum creatinine t0	1	0.0004
Circulating memory CD8+ T cells (%)		Serum creatinine t6	−0.9429	0.0167
Circulating total CD8+ T cells (#)		Serum creatinine t12	0.9276	0.0167
Circulating monocytes (#)		Serum creatinine t12	0.7545	0.0377
Circulating memory CD4+ T cells (#)		Serum creatinine t24	−1	0.0167
Circulating memory CD4+ T cells (#)		Serum creatinine t36	−0.9747	0.0333
Circulating total CD8+ T cells (#)		GFR t0	−0.9286	0.0067
Tissue cytotoxic/Treg ratio		GFR t0	−0.7197	0.0342
Circulating Th17 cells (%)		GFR t6	0.8986	0.0278
Blood Th17/Treg ratio		GFR t6	0.8986	0.0278
Circulating monocytes (#)		GFR t6	−0.7306	0.0476
Circulating monocytes (#)		GFR t12	−0.8333	0.0154
Circulating naïve CD4+ T cells (%)		GFR t36	1.0000	0.0167
Circulating naïve CD4+ T cells (%)		GFR t36	1.0000	0.0167
Circulating naïve CD8+ T cells (%)		Proteinuria t0	0.7857	0.048
Infiltrating CD4+ T cells (%)		Proteinuria t0	0.7668	0.0254
Circulating TCRgd2+ T cells (%)		Proteinuria t6	0.7857	0.048
Circulating monocytes (%)		Proteinuria t6	0.8095	0.0218
Circulating monocytes (#)		Proteinuria t12	0.7619	0.0368
Circulating total CD3+ T cells (%)		Proteinuria t24	−0.9747	0.0333
Circulating Th17 cells (%)		Proteinuria t36	−0.9747	0.0333
Circulating Th17 cells (#)		Proteinuria t36	−0.9747	0.0333
Blood Th17/Treg ratio		Proteinuria t36	−0.9747	0.0333
Tissue cytotoxic/Treg ratio		Proteinuria t36	−0.8407	0.0444
Circulating TCRgd2+ T cells (%)		Time to complete remission	0.8469	0.0238
Circulating TCRgd2+ T cells (#)		Time to complete remission	0.7748	0.0492
Circulating monocytes (%)		Time to complete remission	0.8368	0.0072
Circulating monocytes (#)		Time to complete remission	0.8201	0.0095

## Abbreviations

CTL: Cytotoxic T lymphocyte
GFR: Glomerular filtration rate
IFN: Interferon
MCD: Minimal change disease
MDRD: Modified diet in renal disease
MAIT: Mucosal-associated invariant T
NK: Natural killer
NKT: Natural killer T
SGH: Singapore General Hospital
Teff: Effector T
Th: T helper
Treg: Regulatory T.

## Additional Points

*Summary.* Primary minimal change disease is a common cause of idiopathic nephrotic syndrome in adults. Infections, certain environmental factors, and T cell dysfunction with increased levels of a soluble glomerular permeability factor have been proposed to play a central role in the pathogenesis of this condition. Therefore, a dysfunction, or a relative deficiency of Foxp3^+^ regulatory T cells over effector T cells, could be implicated in its immunopathogenesis. We present here a pilot phenotypic analysis of the major T cell subsets infiltrating the kidney and in the peripheral blood of adult patients suffering from primary minimal change disease. An apparent numerical dominance of T helper 17 cells and cytotoxic cells over Foxp3^+^ Treg cells was detected in the renal interstitium in primary/idiopathic minimal change disease, but we had no control normal kidney tissue for comparison. In peripheral blood, on the other hand, we observed an increase in the circulating total CD8^+^ T cells in patients with minimal change disease. These results suggest that in primary/idiopathic minimal change disease, a relative numerical dominance of effector over regulatory T cells can be observed in kidney tissue and peripheral blood. However, larger confirmatory studies are necessary.
